# Team numerical advantage in Australian rules football: A missing piece of the scoring puzzle?

**DOI:** 10.1371/journal.pone.0254591

**Published:** 2021-07-16

**Authors:** Jeremy P. Alexander, Timothy Bedin, Karl B. Jackson, Sam Robertson

**Affiliations:** 1 Institute for Health and Sport (iHeS), Victoria University, Melbourne, Victoria, Australia; 2 Champion Data Pty Ltd, Melbourne, Victoria, Australia; Arizona State University & Santa Fe Institute, UNITED STATES

## Abstract

The primary aim of this study was to determine the relationship between a team numerical advantage during structured phases of play and match event outcomes in professional Australian football. The secondary aim was to quantify how players occupy different sub-areas of the playing field in match play, while accounting for match phase and ball location. Spatiotemporal player tracking data and play-by-play event data from professional players and teams were collected from the 2019 Australian Football League season played at a single stadium. Logistic regression analysed the relationship between total players and team numerical advantage during clearances and inside 50’s. Total players and team numerical advantage were also quantified continuously throughout a match, which were separated into three match phases (offence, defence, and stoppage) and four field positions (defensive 50, defensive midfield, attacking midfield, and forward 50). Results identified an increased team numerical advantage produced a greater likelihood of gaining possession from clearances or generating a score from inside 50’s. Although, an increased number of total players inside 50 was likely associated with a concomitant decrease in the probability of scoring, irrespective of a team numerical advantage. Teams were largely outnumbered when the ball was in their forward 50 but attained a numerical advantage when the ball was in the defensive 50.

## Introduction

Team performance in invasion sports is dependent on outcomes emanating from continual interactions between teammates and opponents [1]. Players are constantly required to regulate their positioning to either generate offensive opportunities in an attempt to score or to preserve defensive stability [2]. Analysis of this information can be used to describe tactical team behaviour and performance outcomes [2, 3]. By examining how players occupy different sub-areas on a playing field at different timescales, an assessment of how players adjust their movement behaviour to increase offensive efficacy or instigate disarray in opposition defensive structures can be determined [2–4]. This may be attained by producing a team numerical advantage by outnumbering the opposing team [2, 3].

In football, successful match outcomes are associated with a team’s capacity to produce a team numerical advantage during attacking phases of play [2] and to maintain defensive stability by ensuring there are additional defending players compared to the opposition [2, 4]. Similar findings were reported in Australian Football (AF) during two 20-minute halves in a 15v15-match simulation drill with players being largely outnumbered in their forward half but maintaining a team numerical advantage in their defensive half [5]. Overall, the total number of players increased based on where the ball was positioned [5]. Greater variation in numerical dominance during the middle sections on a field has also been reported, which may represent that these sub-areas of play are of critical importance in fostering stability and instability [2, 4, 5]. Further, teams who obtained a numerical advantage produced an increased dominant region in the form of a greater area covered by players in comparison to the opposition [3]. Conversely, those that faced a team numerical disadvantage reported decreased dispersion of players across the field and retreated deeper in their defensive half [3].

Notwithstanding, studies that have examined team numerical advantages in football have inferred the match outcome (win/loss) with a team’s aggregated capacity to generate an outnumber over specific regions throughout a match [2]. As such, an incomplete understanding exists between a team numerical advantage and the impact on specific match event outcomes in a continuous manner. Therefore, work is required to determine the relationship between player positioning and match event outcomes that occur continuously throughout a match. Player positioning and performance outcomes can also be influenced by various contextual variables, such as, match phase, ball location, and quality of opposition [6–8]. Thus, contextual variables should be included when assessing how players occupy different sub-areas on a playing field and how this may influence player positioning and match event outcomes [2, 4].

Investigations of similar nature have been undertaken in 15 v 15 match simulation in AF [5] and in small-sided games (SSG) in football [9]. However, research that is representative of professional level match play remains largely absent. Australian football is a sport where teams compete on an oval shaped field (length = ~160 m, width = ~130 m) with 22 players in total, with 18 on the field and 4 on an interchange [10, 11]. During play, teams have the capacity to gain possession of the ball through an intercept, which is possession gained from the opposition, a clearance, which is possession gained from a contested situation (stoppage), or when the opposing team scores a behind [12]. After gaining possession, the ability to score requires a team to transition the ball to enter their attacking 50-metre zone, known as an inside 50 (I50), to execute a shot at goal.

Determining how players occupy different sub-areas on a playing field has supported a greater understanding of tactical team behaviour in invasion sports. However, investigations that assess the relationship between a team numerical advantage and match event outcomes are yet to be reported in professional match play. In addition, research examining how players occupy different sub-areas on a playing field, whilst accounting for contextual variables, such as match phase and ball location also remains largely absent. Therefore, the primary aim of this study was to determine the relationship between a team numerical advantage during structured phases of play and match event outcomes in Australian football. The secondary aim was to quantify how players occupy different sub-areas of the playing field in match play, while accounting for match phase and ball location.

## Materials and methods

### Data collection and analysis

**Fig 1:**
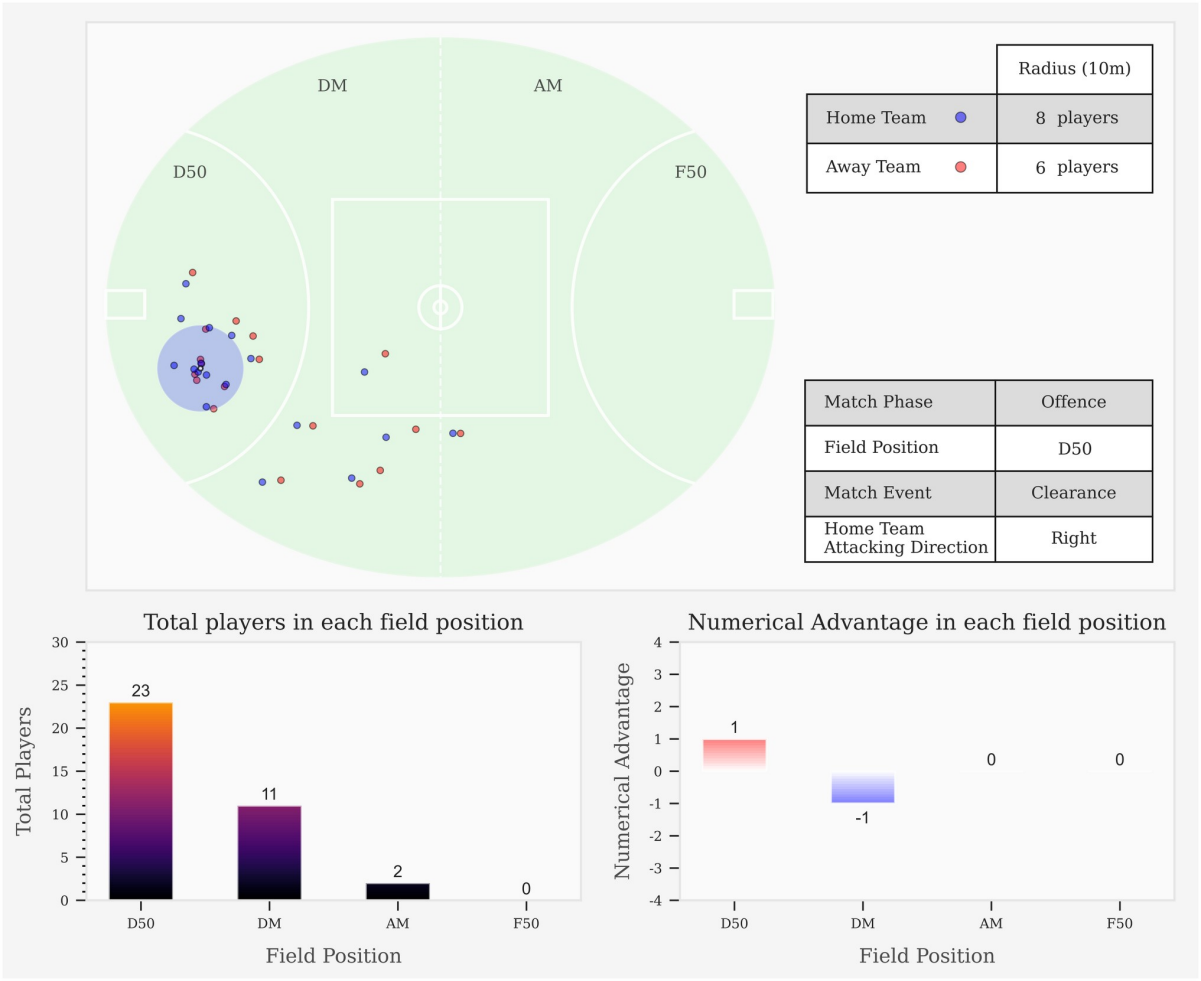
Total count of players for both teams in each field position (bottom left subplot). Team numerical advantage for the Home Team in each field position (bottom right subplot). Total count of players for each team within 10 m radius surrounding ball location. D50, Defensive 50; DM, Defensive Midfield; AM, Attacking Midfield; F50, Forward 50.

Ethical clearance was granted by the University Human Research Ethics Committee (application number HRE20-172). Informed written consent is a part of a wider contractual agreement with the participants and the governing body. Data were collected from the 2019 Australian Football League (AFL) competitive season. To ensure consistency with field dimensions and tracking coordinates, 12 matches were selected from a single stadium (Marvel Stadium, Melbourne, Australia) that took place on an oval shaped ground using dimensions 159.5 m x 128.8 m (length x width). Twelve of the league’s 18 teams were represented in the data, with no team having more than six matches included. To simplify data processing, facilitate visualisation and understanding of results, the teams were labelled Home Team and Away Team for each match (see Fig 1). Matches were undertaken with four 20-min quarters with breaks interspersed between periods. Data for each match were collected using 10 Hz local-positioning system (LPS) devices for all 44 participants (Catapult Optimeye S5, Catapult Innovations, Melbourne, Australia). The devices were housed in a sewn pocket in the jersey that is located on the upper back. Periods of play that lost the positioning of one or more players were omitted.

Play-by-play match event data notated the action of the player, which was manually recorded by a statistical provider to the nearest tenth of a second (Champion Data, Pty Ltd., Melbourne, Australia). Previous investigations have assessed the validity and reliability of similar match event data and reported very high levels (ICC range = 0.947–1.000) of agreement [13]. Positional data were synchronised with match event data using the unix timestamps present in both datasets [14], which was used to infer ball position. Field position of the ball was separated into four zones (defensive 50; D50, defensive mid; DM, attacking mid; AM, forward 50; F50) by the two 50 m arcs and the centre of the ground (see Fig 1), which is conventional for AF research and statistical providers [12, 15, 16]. The impact on match play was determined for three match events–clearances, intercepts, and I50s (entries into the F50), with terminology provided in Table 1. Clearances and intercepts distinguished how teams gained possession of the ball and I50’s were associated with potential for scoring attempts.

**Table 1:** Definitions of commonly reported match events by Champion Data.

Match Event	Definition
Intercept	Gaining possession of the ball directly from an opposition player
Turnover	Losing possession of the ball to an opposition player. One team’s turnover is the other team’s intercept
Clearance	Clearing of the ball out of a stoppage (congested) situation to the advantage of one’s team
Inside 50	The act of running or passing the ball into the 50 m arc at the team’s attacking end of the field
Rebound 50	The act of running or passing the ball outside the 50 m arc at the team’s defensive end of the field
Possession	When a player obtains the ball with adequate time to dispose of it
Goal	A score worth 6 points. Achieved when a player kicks the ball through the two large goalposts without touching the post or any player
Behind	A score worth 1 point. Achieved if a player kicks the ball between the goal post and behind post, hits the goal post, or being touched by a player before passing between goalposts
Disposal	Passing the ball by either a kick or handball

Clearances and intercepts were recorded in each field position. Clearances are obtained from a stoppage encompassing two teams. As such, the clearance outcome in a certain field position was reflected in the respective field position for the opposing team. Intercepts that were obtained from possession lasting less than five seconds were removed from the analysis, as it may not allow adequate time for teams to execute predetermined attacking and defending formations, which may produce turnovers of possession independent of player positioning. Inside 50’s were recorded when teams transitioned the ball into their attacking 50-metre zone. Intercepts and clearances that resulted in a free kick or shots from outside 50 were removed from the investigation, as player positioning may have a limited influence on the outcome. Clearances that resulted in a repeat stoppage, occurring when no team clears the ball from stoppage area, were removed from the study. Clearances that were obtained from centre bounces were also removed, as teams are restricted to four players only in this situation, eliminating the ability to generate a team numerical advantage. Altogether, a total of 831 intercepts, 471 clearances, and 1032 I50’s were recorded.

The total number of players and team numerical advantage inside the four field positions for both teams were assessed for each point in time. Team numerical advantage was recorded in the designated field position when assessing intercepts. The total number of players and team numerical advantage within the F50 was used when assessing the I50 outcome. A 10 m radius surrounding the position of the ball at each point in time was used to measure team numerical advantage and total players during stoppages to measure the clearance outcome. The team numerical status (*Ns*) for the Home Team ($N_{S}^{H}$) and Away Team ($N_{S}^{A}$) was assessed via the following rule:

$$Ns=N_{S}^{H}-N_{S}^{A}.$$

The interaction between total players and team numerical advantage on the scoring outcome of I50’s was assessed through a partial dependence plot (PDP), which is a technique to understand the relationship between features of the model on the outcome. Put simply, partial dependence plots show how each variable affects the model’s predictions.

Match phase was determined via which team had possession of the ball (offence, defence or stoppage). The offensive phase was recorded from when a team won possession from either a clearance, intercept or after the opposing team scored a behind [12]. The offensive phase continued until the team lost possession of the ball to the opposing team or the ball went out of play [17]. The defensive phase was recorded when the opposing team was in offence. If neither team had possession of the ball, for example, when the officiating umpire returned the ball to play, the phase was considered to be in stoppage until a team cleared the ball from this area. Periods where the ball was out of play, for example, when there was a break between periods of play, when the umpire had the ball before a stoppage, and after scores were excluded from the investigation.

### Statistical analysis

Binary logistic regressions were used to determine the relationship between a team numerical advantage, total players and match event outcomes. Specifically, match events included clearances, in each field position, and inside 50’s (entries into the F50 only). Thus, four logistic regressions considered clearance outcome (Team Clearance Win = 1, Team Clearance Loss = 0) as the dependent variable in each field position (D50, DM, AM, F50). A fifth logistic regression considered I50 outcome (Score = 1, Defending Team Rebound 50 or Stoppage = 0) as the dependent variable. Data were segmented into a test-train split 80% and 20% [18]. Odds ratios (OR) and associated 95% confidence intervals (95% CI) were determined to provide a standardised measure of the influence of each variable included in the models [19], which indicate the effect of each feature in the estimated odds of the outcome [20]. The discriminant capacity of each logistic regression model was assessed by determining the receiver operating characteristic (ROC) curve to display the true positive rate (sensitivity) compared to the false positive rate (specificity) [21]. An area under the curve (AUC) was assessed with an AUC of 1, or 100%, demonstrating perfect discriminant capacity [22]. The correct classification (accuracy) of each model was calculated from the sensitivity rate and the specificity rate. The interaction between total players and team numerical advantage on the I50 outcome was also assessed via a partial dependence plot [23]. The partial function ${\hat{\hat{f}}}_{xs}$ was assessed by the mean prediction of the independent variable [23], also known as Monte Carlo method:

$${\hat{\hat{f}}}_{xs}\left(x_{s}\right)=\frac{1}{n}\sum_{i=1}^{n}\hat{\hat{f}}(x_{s},x_{C}^{\left(i\right)})$$

Where, *x*_*s*_ are the features for which the partial dependence function is plotted and *x*_*C*_ are the additional features used in the logistic regression model $\hat{\hat{f}}$. All visualisations were processed using the computational package Python version 3.2 with *Spyder*, which is part of the Anaconda software suite (www.python.org). Analyses were undertaken using in the Python programming language, using the SciPy and Scikit-learn packages.

## Results

### Relationship between a team numerical advantage and match event outcomes

**Fig 2:**
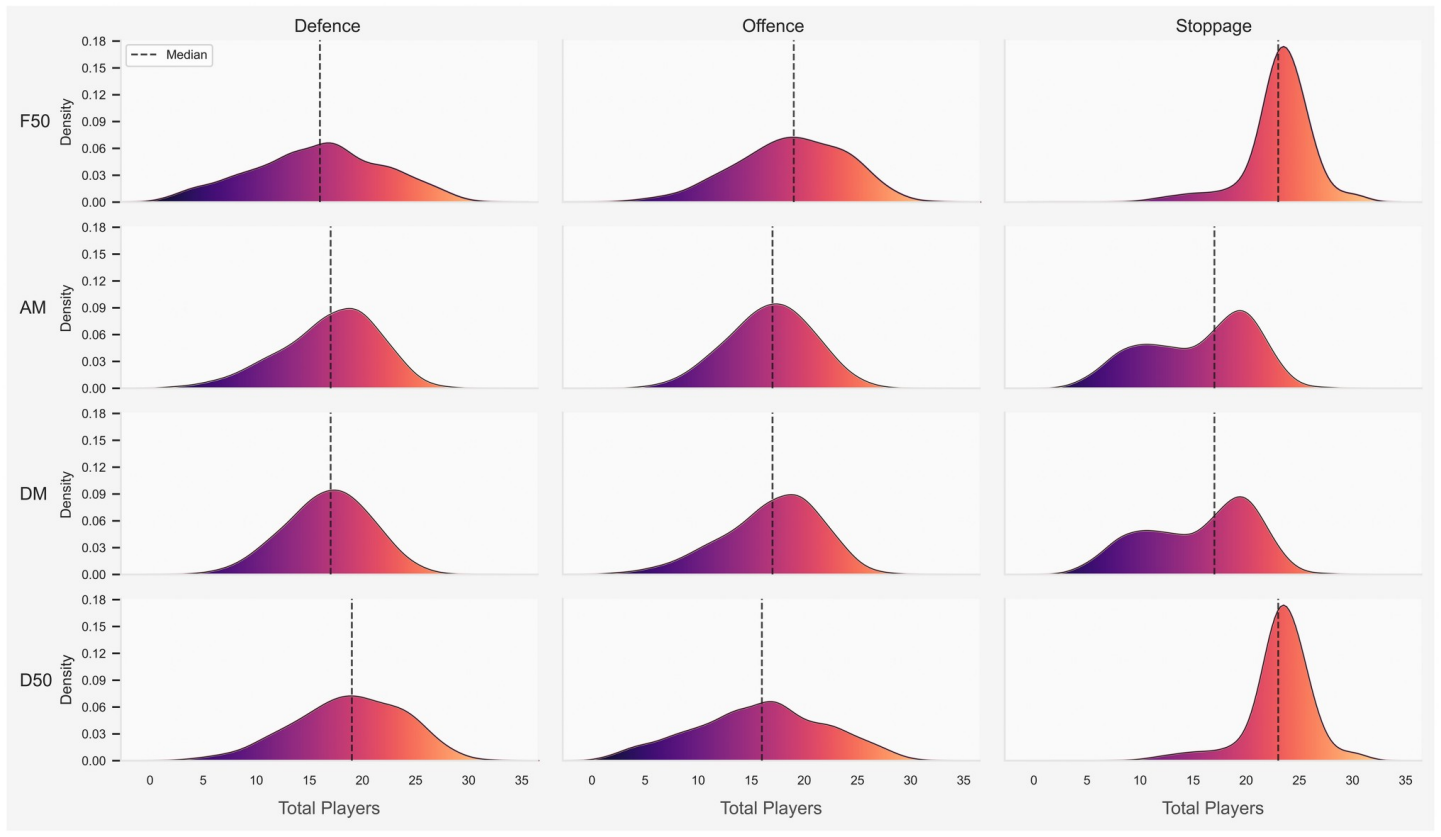
Total count of intercepts in each field position for each team numerical combination.

The total count of intercepts in each field position for each team numerical combination (numerical advantage, even numbers, numerical disadvantage) is displayed in Fig 2. The majority of intercepts were obtained in the D50 and DM with an even number of players or a team numerical advantage.

**Fig 3:**
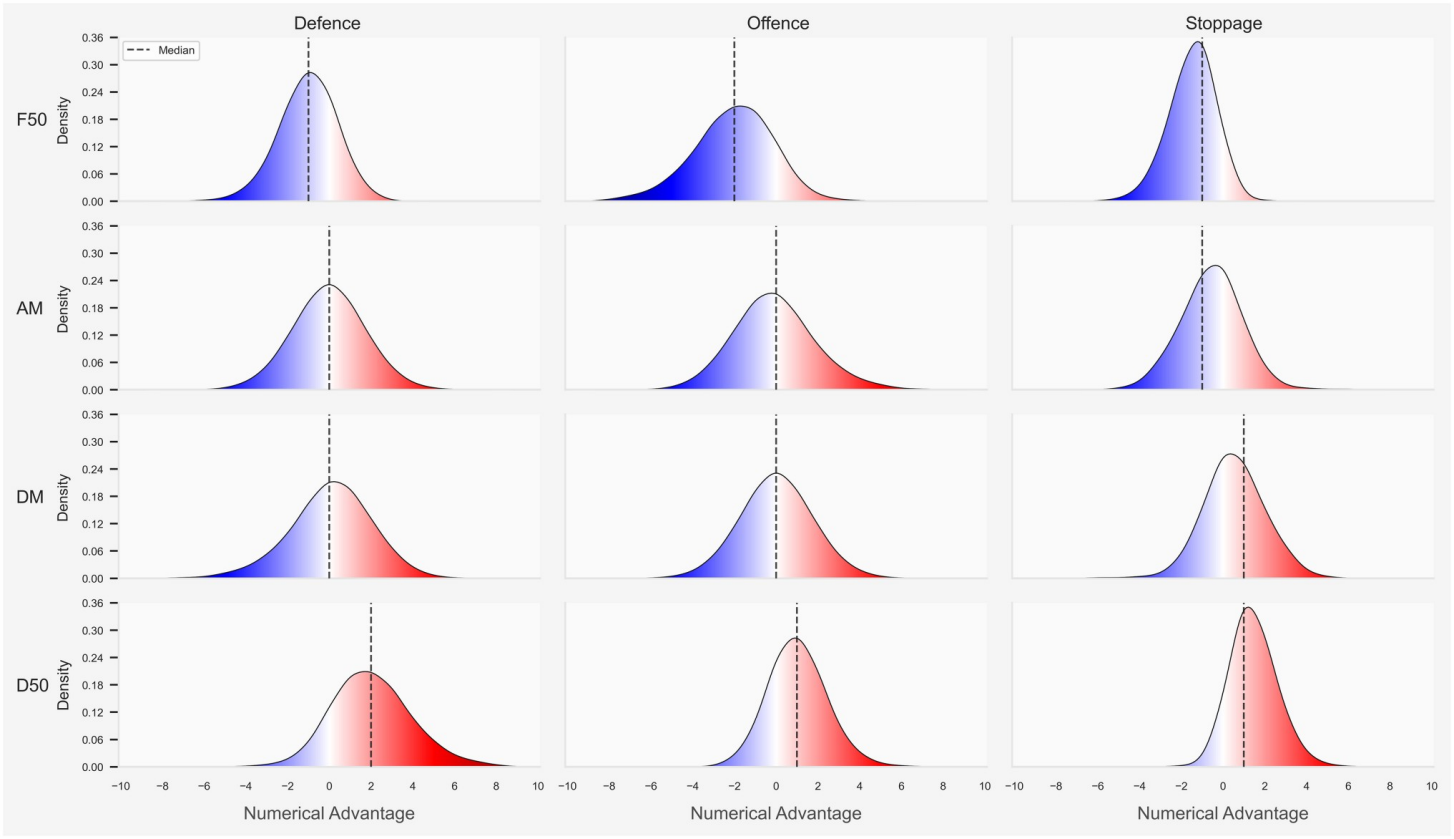
Total count and success rate of clearance outcome in each field position for each team numerical combination. The total count of clearance outcome is displayed on the primary *y*-axis (left). The success rate of clearance outcome corresponds to the secondary *y*-axis (right).

Total count and success rate of clearance outcome in each field position for each team numerical combination is presented in Fig 3. Estimated results from each logistic regression model for obtaining a clearance in each field position are presented in Table 2. Evaluation of each logistic regression model for obtaining a clearance in each field position is presented in Table 3. The majority of clearances were recorded in the AM or DM. The likelihood of generating a clearance increased if a team was able to obtain a numerical advantage at the stoppage, regardless of field position. Nonetheless, teams generally achieved a numerical advantage in the D50, forcing a disadvantage in the opponents’ F50. Team numerical advantage reported a β-coefficient of 0.686 with a standard error of 0.172 and odds ratio of 1.986 (1.418–2.781; 95% CI) in the D50 and F50. The team numerical advantage in the DM and AM recorded a β-coefficient of 1.452 with a standard error of 0.212 and odds ratio of 4.273 (2.819–6.476; 95% CI). Evaluation of the D50 and F50 model displayed a predictive accuracy, sensitivity, and a specificity of 62%, while the AUC was 69%. The DM and AM model recorded a predictive accuracy or 77%, sensitivity of 64%, specificity of 61%, and AUC of 69%.

**Table 2:** Estimated results relating to each logistic regression model for clearance outcome in each field position.

		β (S.E)	*Ρ*	OR (95% CI)
**D50**	Team Numerical Advantage	0.686 (0.172)	<0.001	1.986 (1.418–2.781)
	Total Players	0.001 (0.067)	0.989	1.001 (0.878–1.141)
**DM**	Team Numerical Advantage	1.452 (0.212)	<0.001	4.273 (2.819–6.476)
	Total Players	0.105 (0.078)	0.176	0.901 (0.774–1.048)
**AM**	Team Numerical Advantage	1.452 (0.212)	<0.001	4.273 (2.819–6.476)
	Total Players	0.105 (0.078)	0.176	0.901 (0.774–1.048)
**F50**	Team Numerical Advantage	0.686 (0.172)	<0.001	1.986 (1.418–2.781)
	Total Players	0.001 (0.067)	0.989	1.001 (0.878–1.141)

*Note*: CI is the 95% confidence interval, β is the beta coefficient, S.E is the standard error, OR is the odds ratio. Statistical significance accepted at ≤0.05.

**Table 3:** Evaluation of logistic regression model for clearance outcome in each field position.

	AUC	Sensitivity	Specificity	Accuracy
**D50**	0.69	0.62	0.62	0.62
**DM**	0.77	0.64	0.61	0.61
**AM**	0.77	0.64	0.61	0.61
**F50**	0.69	0.62	0.62	0.62

*Note*: AUC is the area under the curve.

**Fig 4:**
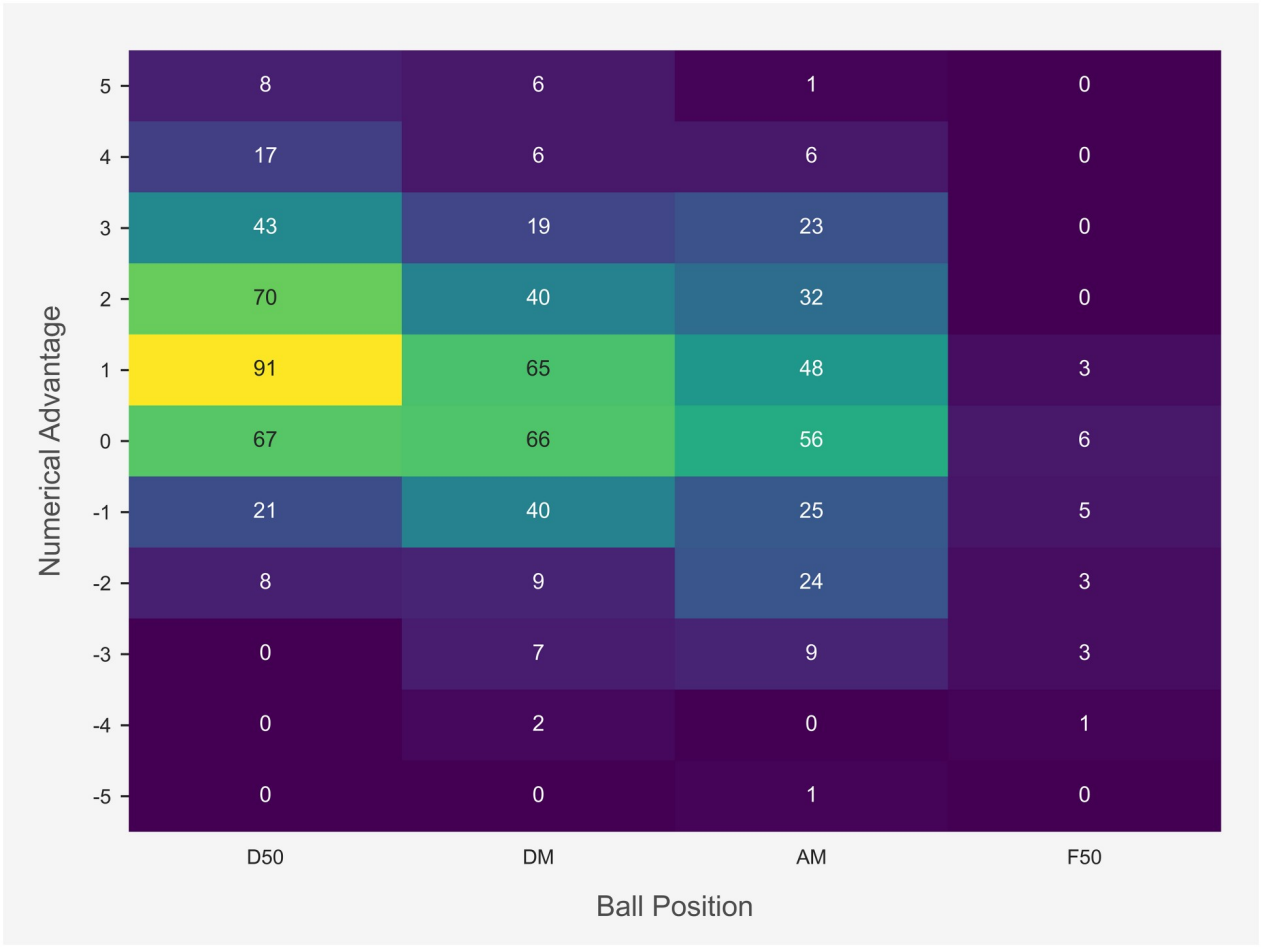
Total count and success rate of I50 outcome for each team numerical combination. The total count of I50 outcome is displayed on the primary *y*-axis (left). The success rate of I50 outcome corresponds to the secondary *y*-axis (right).

**Fig 5:**
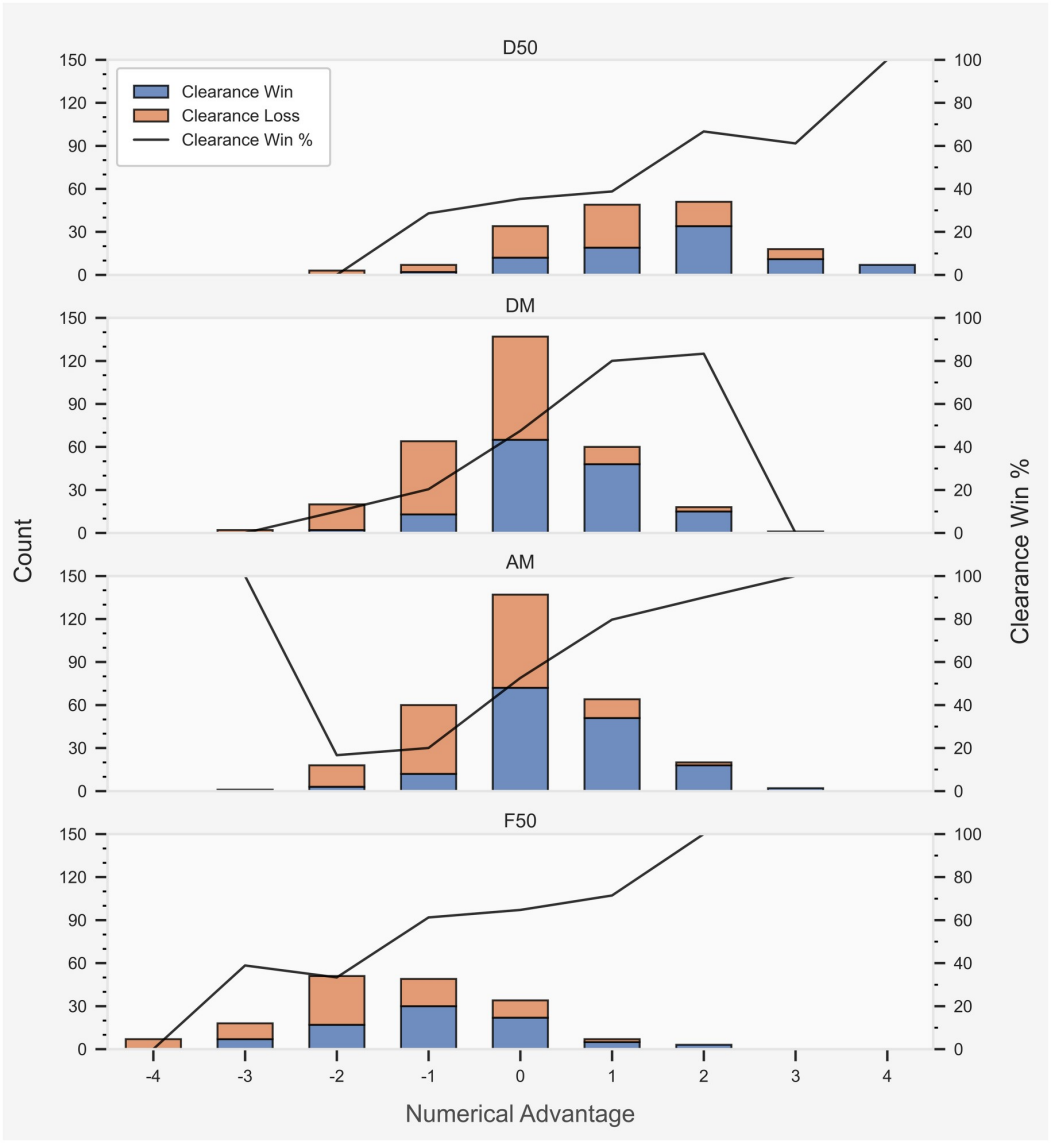
Probability of scoring accounting for the interaction between team numerical advantage and total players.

Total count and success rate of I50 outcome for each numerical combination is displayed in Fig 4. Estimated results from the logistic regression model for I50 outcome are presented in Table 4. Evaluation of logistic regression model for I50 outcome is displayed in Table 5. The interaction between total players and team numerical advantage on the I50 outcome is represented in Fig 5. The predominance of I50 entries confronted a team numerical disadvantage. The likelihood of producing a score was greater with an increased team numerical advantage when entering I50. Specifically, the team numerical advantage reported a β-coefficient of 0.655 with a standard error of 0.082 and odds ratio of 1.925 (1.639–2.261; 95% CI). Evaluation of the I50 model displayed a predictive accuracy or 73%, sensitivity of 72%, specificity of 73%, and AUC of 69%. Interestingly, while increasing the team numerical advantage had a beneficial impact overall, the interaction between total players and team numerical advantage indicates that a greater number of total players I50 is likely associated with a concomitant decrease in the probability of scoring, irrespective of a team numerical advantage (Fig 5).

**Table 4:** Estimated results relating to logistic regression model for I50 outcome.

	β (S.E)	*P*	OR (95% CI)
Team Numerical Advantage	0.655 (0.082)	<0.001	1.925 (1.639–2.261)
Total Players	-0.023 (0.016)	0.139	0.977 (0.947–1.008)

*Note*: CI is the 95% confidence interval, β is the beta coefficient, S.E is the standard error, OR is the odds ratio. Statistical significance accepted at ≤0.05.

**Table 5:** Evaluation of logistic regression model for I50 outcome.

	AUC	Sensitivity	Specificity	Accuracy
**I50**	0.69	0.72	0.73	0.73

*Note*: AUC is the area under the curve.

### Analysis of how players occupy sub-areas on a playing field

**Fig 6:**
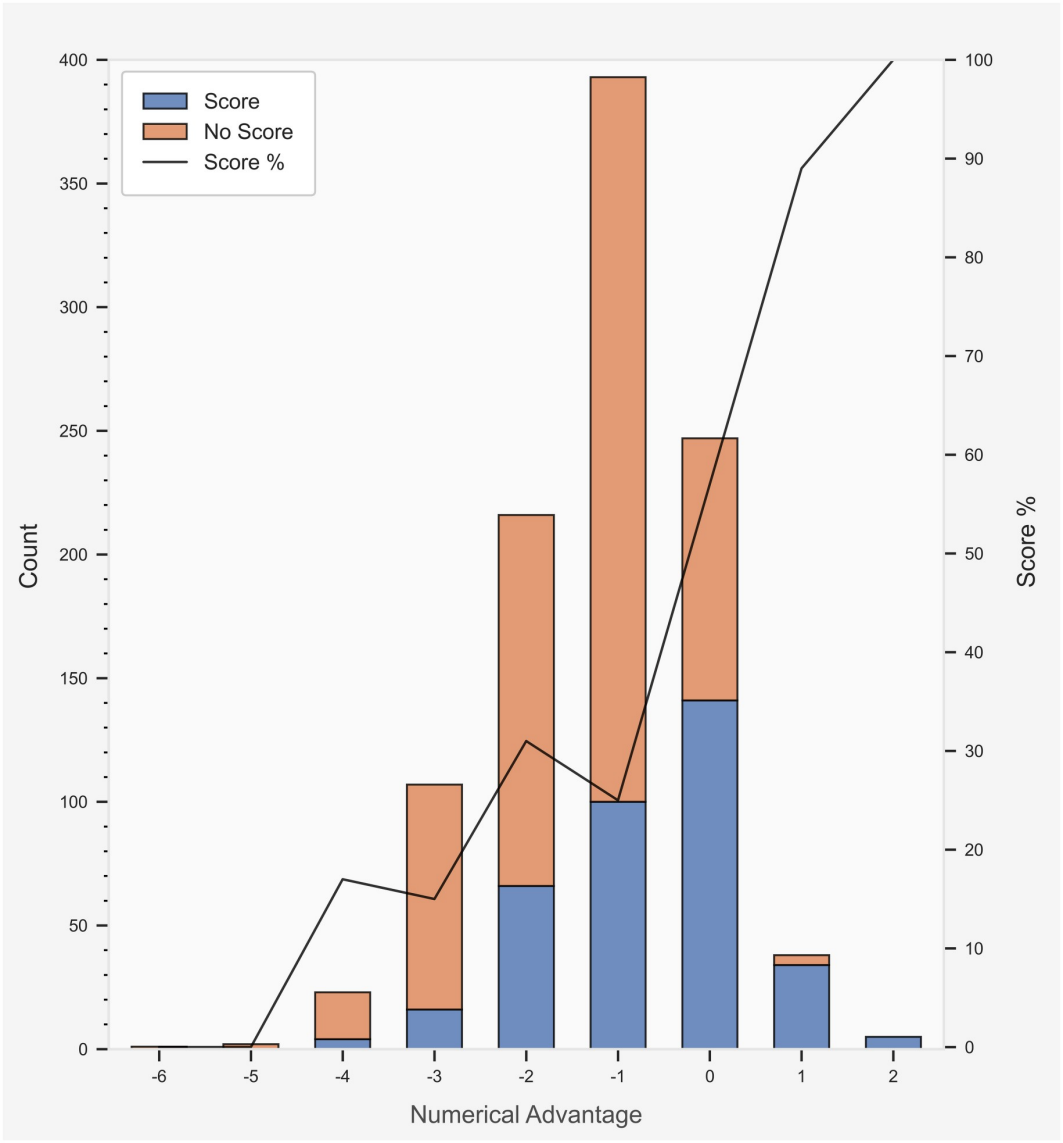
Between match phase and field comparison of the total number of players for both teams. Dotted line represents the median number of total players.

**Fig 7:**
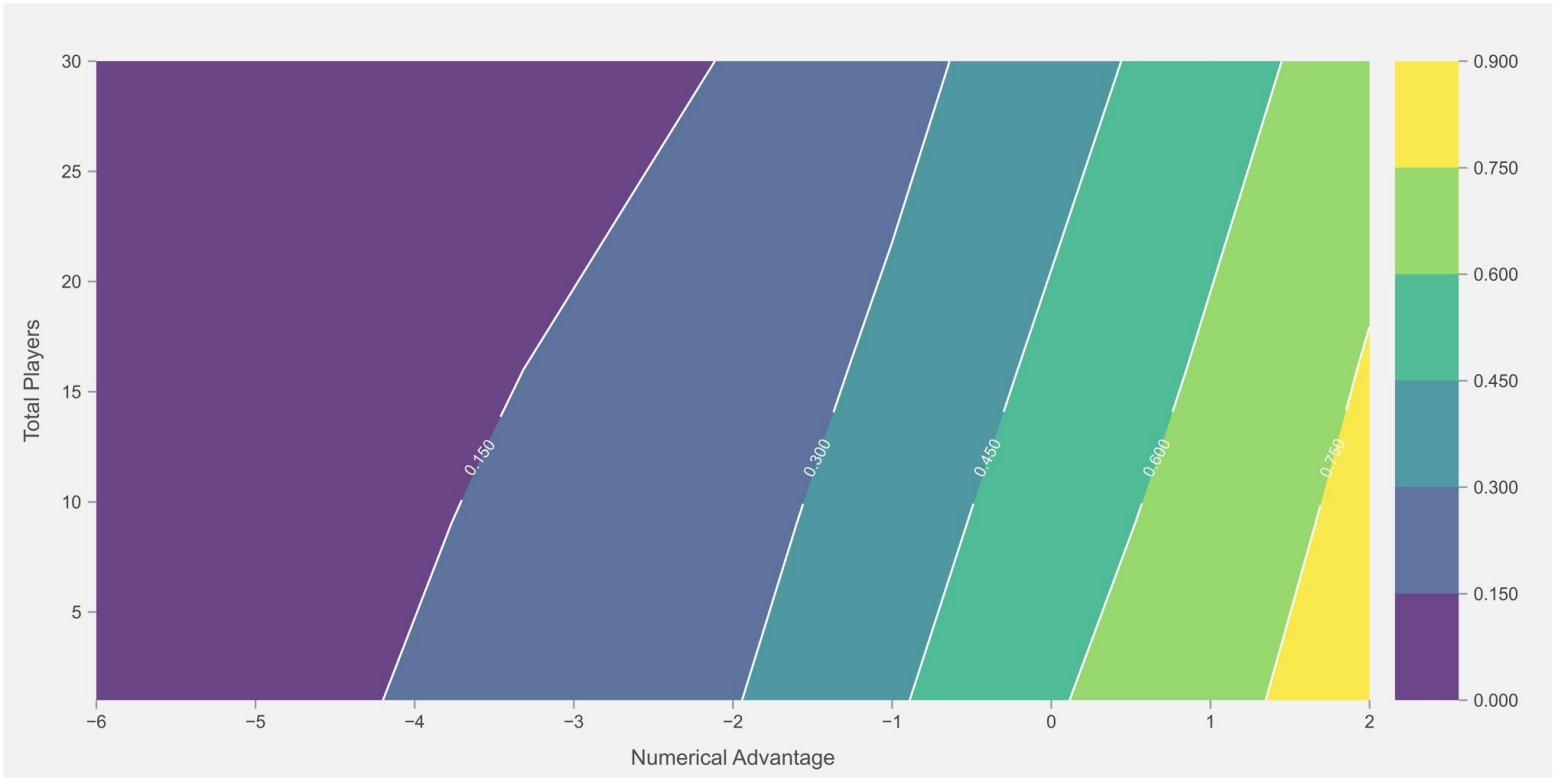
Between match phase and field comparison for team numerical advantage. Dotted line represents the median team numerical advantage.

The distribution of the total number of players and team numerical advantage during each combination of match phase and field position is displayed in Figs 6 and 7 respectively. The total number of players increased based on where the ball was positioned, especially during the stoppage phase in the D50 and F50 and the defensive phase in the D50, with the median number of players ranging from 16 to 24. Teams maintained a numerical advantage in their D50, median of 1 to 2, and encountered a numerical disadvantage in their F50, median of -1 to -2, regardless of match phase.

## Discussion

This study is the first in invasion sports to investigate the relationship between a team numerical advantage and match event outcomes in professional match play. This investigation also furthers the understanding of tactical team behaviour by determining how match phase and ball location influence how players occupy different sub-areas of play during various timescales throughout a match.

An increased team numerical advantage was associated with a greater likelihood of gaining possession of the ball during stoppages or generating a score from preceding I50 entries. Similar to research in match play simulation in AF [5, 17], the total number of players increased based on where the ball was positioned, especially during the stoppage phase in the D50 and F50 and the defensive phase in the D50. It appears that AF players are more nomadic compared to football players, in that, they may not be consigned to a specific role that requires them to be located in a permanent area on a playing surface. The majority of players may contribute to all match phases to create an overall team defence or team offence with the observed number of players in the field position containing the ball regularly reaching 24 to 36 players. This is in contrast to football players who generally perform predetermined roles and therefore occupy fixed regions on a field of play [24]. An increased number of players within a specific region may also impact opposition behaviour [17]. Specifically, increasing the number of defensive players surrounding an attacking team taking a shot at goal is associated with a concurrent decrease in successful scoring attempts [25, 26]. Similarly, findings from this study identified that increasing the number of players I50 is likely associated with a concomitant decrease in the probability of scoring, irrespective of a team numerical advantage.

Teams promoted a numerical advantage in their D50, while confronting a numerical disadvantage in their F50, regardless of match phase. This behaviour may influence the onset of intercepts. Specifically, findings from this investigation may suggest an association between a team numerical advantage and the commencement of an intercept, as intercepts were generally attained in the D50 and DM with a positive team numerical advantage. Teams positioned higher up the field during offence when the ball was in their F50. However, teams rarely obtained even numbers or a positive team numerical advantage in their F50. This behaviour may imply teams employ a somewhat risk averse approach when the ball is inside their F50. Specifically, equalising the number of players or securing a positive team numerical advantage may increase the probability of scoring during offensive sequences. However, it may also instigate greater congestion or expose defensive vulnerabilities if the opposing team were able to gain possession of the ball, thereby, exacerbating counterproductive match event outcomes. As such, when the ball is inside forward 50, teams may be inclined to setup an obstacle or barrier that makes it difficult for the opposing team to rebound from their D50, which may assist in generating repeat F50 entries and opportunities to score.

The fundamental underpinning of invasion sports is the concept of two interconnected yet opposing forces [27, 28]. Teams are continuously challenged to balance their movement behaviour in an attempt to foster opportunities to score, while concurrently maintaining defensive stability. Measuring the specific organisation of player positioning may assist in understanding how teams approach these phases of play. This information may also provide greater insight into the resistive exchange between opposing teams by establishing how individual tactical behaviour interacts to affect the performance outcome. Indeed, particular player positioning may contribute to a team gaining a competitive advantage in certain aspects of match play, whilst concurrently incurring a disadvantage to the opposing team in other match phases. For instance, findings from this study indicates that placing an extra player around stoppages increases the likelihood of gaining possession of the ball to generate a clearance. Teams may allocate more players in these situations to achieve this outcome. However, this may create a performance shortfall elsewhere on the field that is induced by where the player was taken from and more importantly, where the unrestricted opposing player is now situated. For example, if a player is taken from the forward half of the field to assist in stoppage situations, the opposition may have a numerical dominance in their defensive half, which may enable more intercepts in this section of the ground. Other tactical considerations may comprise of enhancing offensive capacity. Specifically, pursuing more direct ball movement to penetrate I50 with reduced defenders will increase the likelihood of generating a score. However, faster ball movement may increase the threat of turning the ball over and being exposed defensively. Conversely, reinforcing defensive stability may be assisted by increasing the team numerical advantage or the total number of players in D50 to restrict opposition scoring or induce turnovers. However, drawing otherwise forward half players to enact this strategy may restrict offensive performance during attacking sequences of play as teams would naturally encounter fewer alternatives to transition the ball towards their attacking end. Crucially, if both teams were to engage additional players in their defensive half, resulting match play may notice an increase in intercepts but a concomitant decrease in scoring. Whilst aforementioned strategies may be supportive in accomplishing singular aspects of match play, it may be prudent to view tactical behaviour in invasions sports in a more holistic interconnected approach [29]. A point of diminishing returns may emerge if there is an overemphasis placed on a certain match phase, which could be detrimental to other aspects of match play. Altogether, a balance between phases of play should be sought to elicit an effective performance output.

This trade-off should be considered by coaches and analysts when assessing individual performance outcomes. Specifically, allocating more players around stoppages may naturally assist midfielders in generating a greater amount of clearances. Similarly, reinforcing defensive structures may be accomplished by assigning extra players in the defensive half to provoke turnovers or restrict scoring. However, forward half players may find it challenging to perform when competing against additional defenders or amongst a large group of players. Expectations of individual performances may need to be tailored to account for such exchanges that accompany varying player roles [30]. For example, when encountering a team that promotes extra defenders, a more equitable representation of a forward’s performance may include the notation of how the opposition was prevented from generating an intercept, rather than solely accounting for traditional performance indicators. Likewise, if a team is confronted with a numerical disadvantage during stoppages, considering the output of the free player(s) is encouraged when assessing individual performances as a whole. Finally, it should be mentioned that player positioning, in and of itself, may not explain all aspects of match event outcomes. Players are still required to execute skilled tasks in order to ascribe a beneficial impact on an aspect of play. Indeed, erroneous skill execution under minimal pressure or an inability to influence ensuing match play may propagate an adverse bearing on a team’s fortune that is independent of player positioning.

Additional research is required that includes an investigation of every club to construct a more accurate representation of how players occupy sub-areas of play and if any variations exist between teams and throughout a season. Athletes should use the same tracking devices to ensure the measurement error is reduced between devices [31]. Further research is also needed to determine the interaction between a team numerical advantage at stoppages and the consequential numerical disadvantage in other field positions and how they affect clearance outcomes. In addition, the length in which these team numerical advantages are maintained, and the corresponding efficacy of its advantage is unknown empirically and requires further investigation. A more nuanced method when assessing I50 outcome may also be informative. This study considered behinds as a beneficial outcome, which may be somewhat oversimplified. Defensive structures may coerce attacking teams into producing a suboptimal shot at goal, which may be considered as effective defensive functioning. Future work may also incorporate a more refined depiction of player positioning and resultant spatial control. This study implemented discrete regions when assessing the team numerical advantage and altering these regions will naturally impact any potential findings. Therefore, a more continuous approach may be appropriate that determines how much space a player can theoretically cover [32], which can be transformed to determine the level of control a player or team exerts over a designated region of play. Solely attributing large portions of arbitrary space to a team that contains an extra player may not adequately attribute the relevant space to a team, thereby overestimating the total influence over that region.

## Conclusion

The results from this study identified that an increased team numerical advantage displayed a greater likelihood of gaining possession of the ball from stoppages or generating a score. Although, an increased number of total players inside 50 was likely associated with a concomitant decrease in the probability of scoring, irrespective of a team numerical advantage. Overall, teams were largely outnumbered when the ball was in their forward 50 but attained a numerical advantage when the ball was in the defensive 50.

## Supporting information

S1 File Numerical advantage and total players during a match. (CSV)
